# Supplementary material for: Slowly evolving dopaminergic activity modulates the moment-to-moment probability of reward-related self-timed movements
Source: eLife. 2021 Dec 23;10:e62583. doi: 10.7554/eLife.62583 (PMC8860451; doi:10.7554/eLife.62583)
Supplement: Figure 1—source data 1. [file elife-62583-fig1-data1.zip › Figure 1/Figure 1--figure supplement 1/Figure 1 supplement 1A/Explanation of Datasets.rtf]

IMPORTANT:For Beginner training days (1-3) and intermediate task, reaction licks are permitted, and the self-timed lick is scored as the first lick occurring after 500 ms after the cueBUT for the histogram, the beginner and intermediate datasets can have up to 2 “first-licks” per trial — the reaction lick and the scored first-lick.To deal with this, we provide a dataset with both the first reaction lick (within 500 ms) and the next first lick after 500 ms.For the Expert task, the only first-lick scored per trial is the first-lick after the cue. Here, you will find there are fewer “reaction” licks because the mouse is penalized for themTimepoints are in SECONDS relative to the cue event
