# Supplementary material for: Slowly evolving dopaminergic activity modulates the moment-to-moment probability of reward-related self-timed movements
Source: eLife. 2021 Dec 23;10:e62583. doi: 10.7554/eLife.62583 (PMC8860451; doi:10.7554/eLife.62583)
Supplement: Figure 2—source data 4. [file elife-62583-fig2-data4.zip › Figure 2--supp-3/Figure 2-figure supplement 3/Explanation of Datasets.rtf]

All original figures provided for each panelTo extract source datapoints, run:line:h = findobj(gca,'Type','line')x=get(h,'Xdata')y=get(h,’Ydata')
