# Supplementary material for: Slowly evolving dopaminergic activity modulates the moment-to-moment probability of reward-related self-timed movements
Source: eLife. 2021 Dec 23;10:e62583. doi: 10.7554/eLife.62583 (PMC8860451; doi:10.7554/eLife.62583)
Supplement: Figure 5—source data 1. [file elife-62583-fig5-data1.zip › Figure 5/C/Explanation of Datasets.rtf]

This folder contains source data for panel CFigure 1CDatasets are included for the Percentage Loss Improvement panel C, rightEach datapoint is a session, units are percentage of additional improvement of Timing-Dependent model over the Timing-Independent modelFigure supplement: original figures provided.To extract source datapoints, run:line:h = findobj(gca,'Type','line')x=get(h,'Xdata')y=get(h,’Ydata')scatter plots:h = findobj(gca,'Type','scatter')x=get(h,'Xdata')y=get(h,’Ydata')
