# Supplementary material for: Slowly evolving dopaminergic activity modulates the moment-to-moment probability of reward-related self-timed movements
Source: eLife. 2021 Dec 23;10:e62583. doi: 10.7554/eLife.62583 (PMC8860451; doi:10.7554/eLife.62583)
Supplement: Figure 5—source data 1. [file elife-62583-fig5-data1.zip › Figure 5/B/Explanation of Datasets.rtf]

This folder contains source data for panel B5-fold cross-validated simulated data is provided as a .mat file.The 300x simulated fit error is provided in a separate .mat file as timeseries of 95% confidence intervals.The vector length of each cell corresponds to the modeled first-lick time. NB that simulations were run with 1500 ms time before the cue, thus a dataset with 2000 samples is for a simulated first-lick at 0.5 s relative to the cue.Open source data and run:figure,for ii = 1:numel(simdata_x)	plot(simdata_x{ii,1}, simdata_y{ii,1}), hold on	plot(simdata_x{ii,1}, CI95_lower{ii,1}, 'k-')	plot(simdata_x{ii,1}, CI95_upper{ii,1}, 'k-')end
