# Supplementary material for: Slowly evolving dopaminergic activity modulates the moment-to-moment probability of reward-related self-timed movements
Source: eLife. 2021 Dec 23;10:e62583. doi: 10.7554/eLife.62583 (PMC8860451; doi:10.7554/eLife.62583)
Supplement: Figure 6—source data 1. [file elife-62583-fig6-data1.zip › Figure 6/Figure 6--figure supplement 2/Explanation of Datasets.rtf]

Original collated statistical object provided containing step and ramp fits across all trials for all sessions.obj.iv: initial variables containing details of the analysisobj.collatedResults: contains data for every session included in analysisobj.analysis: This field is empty in the saved object and holds transient variables when using analysis functions from Github Plotted datapoints can also be extracted from .eps encapsulated objects in folder(Original files panel B: /Users/lilis/Dropbox (MIT)/1 ASSAD LAB/testFiles/testHOST/sessionHOST/CSVfigures/collatedCSV/b5_SNc_13/B5_d13_HIERARCH)Fitting trimmed 500ms after cue, 150ms before lick, 20 traces, 50 rounds computation e/a
