# Supplementary material for: Slowly evolving dopaminergic activity modulates the moment-to-moment probability of reward-related self-timed movements
Source: eLife. 2021 Dec 23;10:e62583. doi: 10.7554/eLife.62583 (PMC8860451; doi:10.7554/eLife.62583)
Supplement: Figure 8—source data 1. [file elife-62583-fig8-data1.zip › Figure 8/Figure 8--figure supplement 1/Explanation of Datasets.rtf]

Julia datafiles saved in CSV format for each animal. Each folder contains model results for each timeslice, along with .eps images to visualize how the data went into the figures.
