# Supplementary material for: Slowly evolving dopaminergic activity modulates the moment-to-moment probability of reward-related self-timed movements
Source: eLife. 2021 Dec 23;10:e62583. doi: 10.7554/eLife.62583 (PMC8860451; doi:10.7554/eLife.62583)
Supplement: Figure 8—source data 1. [file elife-62583-fig8-data1.zip › Figure 8/Figure 8--figure supplement 1/MouseB5-Composite_bl_ts_pkg2_H6_CImax10/00Note on H6 label.rtf]

Data is from mouse B5, but folders have H6 as animal ID secondary to typo at runtime. 
