# Supplementary material for: Slowly evolving dopaminergic activity modulates the moment-to-moment probability of reward-related self-timed movements
Source: eLife. 2021 Dec 23;10:e62583. doi: 10.7554/eLife.62583 (PMC8860451; doi:10.7554/eLife.62583)
Supplement: Figure 8—source data 1. [file elife-62583-fig8-data1.zip › Figure 8/C/3-4-21 Nested DA and Hx w DA alone/__Explanation of Datasets.rtf]

Julia datafiles saved in CSV format. Results from each nested model provided, along with .eps images to visualize how the data went into the figure panels.CI: confidence intervals for each bootstrapped fitdev_explained: deviance explained for each bootstrapped fitth_summary: contains fit weight values and statisticsths: contains the fit weights alonese_ths: standard errors of fit weightsdofs: contains the degrees of freedom for propagating error across sessionsModels: (from leftmost panel to rightmost in Figure 8C)Hx2s_ — history terms from -2s to -1.8s before time tHx2s_1-6s — history terms from -2s to -1.6s before time tHx2s_1-4s — history terms from -2s to -1.4s before time tHx2s_1-2s — history terms from -2s to -1.2s before time tHx2s_1s — history terms from -2s to -1s before time tHx2s_-8s — history terms from -2s to -0.8s before time tHx2s_-6s — history terms from -2s to -0.6s before time tHx2s_-4s — history terms from -2s to -0.4s before time tHx2s_-2s — history terms from -2s to -0.2s before time tDA_ — GCaMP6f measurement at time t is only predictor
