# Supplementary material for: Slowly evolving dopaminergic activity modulates the moment-to-moment probability of reward-related self-timed movements
Source: eLife. 2021 Dec 23;10:e62583. doi: 10.7554/eLife.62583 (PMC8860451; doi:10.7554/eLife.62583)
Supplement: Figure 8—source data 1. [file elife-62583-fig8-data1.zip › Figure 8/D/3-4-21 Full DA Hx and Hazard/__Explanation of Datasets.rtf]

Julia datafiles saved in CSV and .mat formats for each session and animal.COMPOSITE98_haz_hazs.csvCOMPOSITE98_haz_lts.csvCOMPOSITE98_haz_seshCodes.csvhaz_by_mID_compos_COMPOSITE98_haz_2021-03-09_0121.epshaz_results_composite_COMPOSITE98_haz_2021-03-09_0121.epshazbymouse_2021-03-09_0121.mat — the mean fit hazard for each mousehazs_COMPOSITE_2021-03-09_0020.mat — the fit hazard for each sessionIRTbymouse_2021-03-09_0121.mat — the true mean IRT for each mouselts_COMPOSITE_2021-03-09_0020.mat — the lick times for each session — see seshCodes variable for session IDsmeanHaz_2021-03-09_0121.mat — the mean fit hazard across micemeanIRT_2021-03-09_0121.mat — the mean IRT across mice (true data)names_2021-03-09_0121.mat — the names of each mouse, n=12ndp_per_sample_2021-03-09_0020.mat —the minimum number of trials required for fitting, here 50 meeting criteriaplotxs_2021-03-09_0121.mat — vector of timepoints for x-axis of plotseshCodes_COMPOSITE_2021-03-09_0020.mat — session IDs
