# Supplementary material for: Slowly evolving dopaminergic activity modulates the moment-to-moment probability of reward-related self-timed movements
Source: eLife. 2021 Dec 23;10:e62583. doi: 10.7554/eLife.62583 (PMC8860451; doi:10.7554/eLife.62583)
Supplement: Transparent reporting form [file elife-62583-transrepform1.pdf]

eLIFE

1st Floor
24 Hills Road
Cambridge CB2 1JP, UK

P 01223 855340
W elifesciences.org
T @elife

eLife's transparent reporting form

We encourage authors to provide detailed information *within their submission* to facilitate the interpretation and replication of experiments. Authors can upload supporting documentation to indicate the use of appropriate reporting guidelines for health-related research (see EQUATOR Network), life science research (see the BioSharing Information Resource), or the ARRIVE guidelines for reporting work involving animal research. Where applicable, authors should refer to any relevant reporting standards documents in this form.

If you have any questions, please consult our Journal Policies and/or contact us: editorial@elifesciences.org.

Sample-size estimation

- You should state whether an appropriate sample size was computed when the study was being designed
- You should state the statistical method of sample size computation and any required assumptions
- If no explicit power analysis was used, you should describe how you decided what sample (replicate) size (number) to use

Please outline where this information can be found within the submission (e.g., sections or figure legends), or explain why this information doesn't apply to your submission:

This information is found in the Methods section and in the ARRIVE checklist reporting standards details document in further detail.

Replicates

- You should report how often each experiment was performed
- You should include a definition of biological versus technical replication
- The data obtained should be provided and sufficient information should be provided to indicate the number of independent biological and/or technical replicates
- If you encountered any outliers, you should describe how these were handled
- Criteria for exclusion/inclusion of data should be clearly stated
- High-throughput sequence data should be uploaded before submission, with a private link for reviewers provided (these are available from both GEO and ArrayExpress)

Please outline where this information can be found within the submission (e.g., sections or figure legends), or explain why this information doesn't apply to your submission:

eLife Sciences Publications, Ltd is a limited liability non-profit non-stock corporation incorporated in the State of Delaware, USA, with company number 5030732, and is registered in the UK with company number FC030576 and branch number BR015634 at the address 1st Floor, 24 Hills Road, Cambridge CB2 1JP | August 2014

When/how often experiments occurred is detailed in the Methods section and ARRIVE standards details documentation.

The number of replicates for each experiment is indicated in the Results and Figure Legends when each experiment is first introduced, and reproducibility is described in the ARRIVE standards details documentation. Biological replicates in this study are the individual mice; technical replicates are behavioral sessions and individual trials completed by the same mouse. The type of replicate is made explicit in each figure panel/legend and text description in the Results section (e.g., 4 mice, 5 sessions each).

All data are publicly available in our Data Repository. Source Data files are provided for all figures. A text-file annotation note is included with each Source Data file with instructions for the user.

Outliers were included in analysis. Figures show individual data points as well as summary statistics so that outliers may be seen. Robust statistics are reported (e.g., median).

Inclusion/exclusion criteria are described in the Methods section and with the ARRIVE checklist reporting standards details document.

There was no sequencing data in this study. (N/A)

Statistical reporting

- Statistical analysis methods should be described and justified
- Raw data should be presented in figures whenever informative to do so (typically when N per group is less than 10)
- For each experiment, you should identify the statistical tests used, exact values of N, definitions of center, methods of multiple test correction, and dispersion and precision measures (e.g., mean, median, SD, SEM, confidence intervals; and, for the major substantive results, a measure of effect size (e.g., Pearson's r, Cohen's d)
- Report exact p-values wherever possible alongside the summary statistics and 95% confidence intervals. These should be reported for all key questions and not only when the p-value is less than 0.05.

Please outline where this information can be found within the submission (e.g., sections or figure legends), or explain why this information doesn't apply to your submission:

All statistical methods are described in detail in the Methods section under section subheadings for each experiment.

Raw data is shown in all figures **overlayed with** summary statistics like boxplots or average traces where appropriate.

The Results and Figure Legends specify the statistical test used, N, center definition, dispersion/precision measures, multiple test corrections, and effect size when the results of each experiment are first reported.

Exact p-values shown whenever possible in addition to confidence intervals (in Results and in Figure panels and legends). Source data contains exact p-values for each experiment.

(For large datasets, or papers with a very large number of statistical tests, you may upload a single table file with tests, Ns, etc., with reference to sections in the manuscript.)

Group allocation

- Indicate how samples were allocated into experimental groups (in the case of clinical studies, please specify allocation to treatment method); if randomization was used, please also state if restricted randomization was applied
- Indicate if masking was used during group allocation, data collection and/or data analysis

Please outline where this information can be found within the submission (e.g., sections or figure legends), or explain why this information doesn't apply to your submission:

This information is found in the Results section when results are first mentioned for each experiment as well as in the ARRIVE guidelines reporting details documentation.

Additional data files ("source data")

- We encourage you to upload relevant additional data files, such as numerical data that are represented as a graph in a figure, or as a summary table
- Where provided, these should be in the most useful format, and they can be uploaded as "Source data" files linked to a main figure or table
- Include model definition files including the full list of parameters used
- Include code used for data analysis (e.g., R, MatLab)
- Avoid stating that data files are "available upon request"

Please indicate the figures or tables for which source data files have been provided:

eLife Sciences Publications, Ltd is a limited liability non-profit non-stock corporation incorporated in the State of Delaware, USA, with company number 5030732, and is registered in the UK with company number FC030576 and branch number BR015634 at the address 1st Floor, 24 Hills Road, Cambridge CB2 1JP | August 2014

Source data files are provided for **all figures**.

All neural/behavioral data supporting the findings of this study are publicly available (https://www.dropbox.com/sh/g671nzziba405e3/AAByufom8BYs7PctECCbdiPua?dl=0). Repository to be moved to Zenodo/FigShare within the month of September, 2020.

Data included in the repository include all original single session datasets (behavioral and neural datasets) formatted for ready use in Matlab. When used with the code in our Github repository (http://www.github.com/HarvardSchoolofMouse), all experimental results can be replicated.

Detailed model parameterization is provided in the Methods section.

All MatLab code and necessary dependencies for running behavior and analysis are available via the GitHub link in our Code Availability statement (http://www.github.com/HarvardSchoolofMouse)

Subject baseline data is tabulated in the reporting standards documentation (Baseline Subject Data).
